# Quantification of Carbon and Phosphorus Co-Limitation in Bacterioplankton: New Insights on an Old Topic

**DOI:** 10.1371/journal.pone.0099288

**Published:** 2014-06-11

**Authors:** Irene Dorado-García, Juan Manuel Medina-Sánchez, Guillermo Herrera, Marco J. Cabrerizo, Presentación Carrillo

**Affiliations:** 1 Instituto del Agua, Universidad de Granada, Granada, Spain; 2 Departamento de Ecología, Facultad de Ciencias, Universidad de Granada, Granada, Spain; Instituto de Biologia, Brazil

## Abstract

Because the nature of the main resource that limits bacterioplankton (e.g. organic carbon [C] or phosphorus [P]) has biogeochemical implications concerning organic C accumulation in freshwater ecosystems, empirical knowledge is needed concerning how bacteria respond to these two resources, available alone or together. We performed field experiments of resource manipulation (2×2 factorial design, with the addition of C, P, or both combined) in two Mediterranean freshwater ecosystems with contrasting trophic states (oligotrophy vs. eutrophy) and trophic natures (autotrophy vs. heterotrophy, measured as gross primary production:respiration ratio). Overall, the two resources synergistically co-limited bacterioplankton, i.e. the magnitude of the response of bacterial production and abundance to the two resources combined was higher than the additive response in both ecosystems. However, bacteria also responded positively to single P and C additions in the eutrophic ecosystem, but not to single C in the oligotrophic one, consistent with the value of the ratio between bacterial C demand and algal C supply. Accordingly, the trophic nature rather than the trophic state of the ecosystems proves to be a key feature determining the expected types of resource co-limitation of bacteria, as summarized in a proposed theoretical framework. The actual types of co-limitation shifted over time and partially deviated (a lesser degree of synergism) from the theoretical expectations, particularly in the eutrophic ecosystem. These deviations may be explained by extrinsic ecological forces to physiological limitations of bacteria, such as predation, whose role in our experiments is supported by the relationship between the dynamics of bacteria and bacterivores tested by SEMs (structural equation models). Our study, in line with the increasingly recognized role of freshwater ecosystems in the global C cycle, suggests that further attention should be focussed on the biotic interactions that modulate resource co-limitation of bacteria.

## Introduction

In aquatic ecosystems, bacterioplankton is regulated by different factors [1], including: abiotic such as temperature [2]–[4] or inorganic and organic nutrient sources [5], [6]; and biotic, such as predation [7]–[9]. In turn, bacterioplankton regulate such ecological processes as water quality, atmospheric composition, nutrient cycling [10], and the breakdown of organic matter [11]. Specifically, heterotrophic bacteria remineralize nutrients [1], [12], [13] via general or specialized biogeochemical pathways and, in turn, transfer them to high trophic levels through the microbial loop [14], [15]. In this regard, bacteria convert dissolved organic carbon (DOC) to biomass throughout bacterial production (BP) and/or oxidize it to CO_2_ through bacterial respiration (BR) [16].

Depending on relative availability of organic carbon (C) and nutrients (e.g. P), bacteria can be C or P limited. The nature of the resource that limits bacteria has biogeochemical implications concerning organic C accumulation in freshwater ecosystems. Thus, when bacteria are limited by C having enough inorganic nutrients available, bacteria consume labile C for growth, while semi-labile C tends to accumulate in the surrounding waters [12], [17]. Contrarily, if bacteria are limited by inorganic nutrients, e.g. P, the labile share of the C pool also accumulates in the ecosystem. This connects with the “malfunctioning microbial loop” hypothesis of [18], proposing an accumulation of degradable C because food-web interactions restrict bacterial growth. Controversy persists concerning resource limitation of bacteria in aquatic ecosystems. On the one hand, bacteria are presumed to be P limited in oligotrophic lakes [19], [20] and/or in those with high DOC:P ratios [21], despite that bacteria have a greater affinity for P than do phytoplankton in P-poor aquatic ecosystems [18], [22], [23]. On the other hand, some studies have shown that bacteria are C limited, preferring autochthonous C provided by algae [24]–[26], even though they could use other C resources such allochthonous DOC [24], [27], old DOC (i.e. not recently produced [25], [28]) or semi-labile DOC. Several studies show, however, a prevalence of mineral-nutrient and organic carbon co-limitation in aquatic microbial communities, deduced from stronger responses to combined resources than to single-nutrient additions [13], [26].

Most studies using the nutrient manipulation of bacteria in freshwater ecosystems (increasing C and P) have been conducted in oligotrophic ecosystems because their nutrient-limited condition makes them sensitive to nutrient inputs, and they can act as early-warning ecosystems for eutrophication due to human activities [20], [26]. Thus, studies performed in freshwater ecosystems involve arctic and alpine lakes [20], [29], tropical regions [26], [30], [31] or humic lakes [13], whereas inland waters of Mediterranean region have received less attention. Noticeably, the Mediterranean ecoregion shows a broad intersystem trophic gradient for inland waters, not only throughout the entire ecoregion, but also locally [32]. This has been promoted by traits such as scarceness of vegetation and developed soil on watersheds [33], the high frequency of extreme weather events [34], [35], and millennia of land use (i.e. farming and cattle raising). Moreover, those ecosystems tend to be more autotrophic with greater eutrophy, as has been reported based on estimated BR:PP ratio values [32], probably related to the restricted terrestrial C input [36]. Likewise, the Mediterranean freshwater ecosystems undergo intense and irregular Saharan dust loads that constitute notable inputs of P [37]–[39] and of soil-derived organic components to these water bodies [39], [40]. These resource inputs could alter the microbial community and its functioning [38], [41] and hence biogeochemical cycles in which they are involved [10], [42].

It has been proposed that bacterial P limitation would be more frequent towards oligotrophy, C limitation towards eutrophy, and combined C and P limitation in a wide variety of eutrophic-oligotrophic ecosystems [21]. This trend is partially supported by an across-system study [43] where the effect of P additions diminished towards eutrophy, while the effect of added organic carbon did not correlate with the trophic state. Controversy remains, however, because other studies (e.g. [23]) have found that bacterial production was not stimulated by mineral nutrient (P and N) addition in oligotrophy. Regarding the possible relationship between resource co-limitation and the trophic state, [32] found that P and C co-limited bacteria towards the more eutrophic systems.

Several studies focussing on the co-limitation in primary producers have tried to categorize the kinds of co-limitation based on several mathematical approaches [44]–[47]. While co-limitation has been generally defined as the higher response to combined nutrient additions than to single ones, some studies (e.g. [44]–[47]) have distinguished different kinds of co-limitation. These have been generically based on whether responses to joint factors were higher (synergistic or super-additive co-limitation), equal (additive co-limitation) or lower than the sum of single responses (antagonistic or sub-additive co-limitation). However, these mathematical approaches have not been applied to studies examining co-limitation of bacteria. A unified classification of these kinds and their terminology is still lacking and, moreover, there is scant theory to predict the prevailing kinds of co-limitation of bacteria expected in ecosystems with different trophic states.

Few studies dealing with nutrient limitation of bacteria measure both structural (e.g. biomass) and functional (BP and BR) response variables. In fact, hardly any studies have measured BR in this context due to the difficulties of separating the bacterial fraction from the rest of planktonic community [13]. Therefore, BR has often been estimated from BP values using empirical models (e.g. [32], [48]), despite of troubles posed by the universal application of these estimated measurements.

Because the nature of the resource (C, P) that limits bacteria has biogeochemical implications concerning organic C accumulation in freshwater ecosystems, it is important to increase the knowledge concerning how bacteria respond to C and/or P. Accordingly, we used an experimental approach to evaluate whether the simultaneous addition of C and P (co-) limited bacteria by measuring their responses to C and P addition alone or in combination in two contrasting ecosystems (in terms of the trophic state) in the Mediterranean region. The two ecosystems chosen were a eutrophic reservoir (Cubillas, Granada, Spain) and an oligotrophic high-mountain lake (La Caldera, Sierra Nevada National Park, Spain), two ecosystems that provide major services to surrounding human populations.

Based on patterns of resource limitation reported formerly by our research team [32] for the Mediterranean region, and the positive correlation between bacterial abundance and DOC concentration found in alpine lakes [39], we hypothesise that the bacterial community would be C limited in the oligotrophic high-mountain ecosystem, but limited mainly by P in the eutrophic ecosystem. In addition, we expect that, in both study ecosystems, bacterial responses to joint C and P additions would be more intense (synergistic), according to the generalized resource co-limitation found in aquatic ecosystems, at least for primary producers [46], [47], [49]. In this regard, the use of a modified index based on [47] enabled us to quantify the type of resource co-limitation in bacterioplankton when simultaneous resources, organic carbon (energy substrate) and phosphorus (mineral nutrient) were added.

## Materials and Methods

### Study Area

Nutrient-manipulation experiments were conducted in two Mediterranean ecosystems with contrasting trophic states (Cubillas and La Caldera) located in southern Spain. Cubillas reservoir, of medium size, has a capacity of 21×10^6^ m^3^ and a surface area of 2.0 km^2^ [40]. The water column is characterized by low Secchi depth (<0.5 m), and high values of chemical and biological variables (total nitrogen [TN] >2000 mg m^−3^; total P [TP] >25 mg m^−3^; chlorophyll *a* [Chl a] >30 mg m^−3^; [40]) inherent of eutrophic trophic state [50]. Cubillas reservoir was also selected because it provides cultural ecosystem services to the surrounding urban populations.

On the other hand, La Caldera is a fishless oligotrophic high-mountain lake with a total surface area of 0.02 Km^2^ located in Sierra Nevada National Park (southern Spain) at an elevation of 3050 m.a.s.l. The water is highly transparent (Secchi’s visibility for the entire water column) and registers low values of chemical and biological variables (TP<5 mg m^−3^; Chl a <2 mg m^−3^; DOC ∼0.5 mg C L^−1^; [51], [52]), inherent of oligotrophic trophic state [50].

Research permits for this study were provided by Agencia de Medio Ambiente y Agua (Consejería de Medio Ambiente y Ordenación del Territorio, Junta de Andalucía, Spain) for Cubillas, and by Sierra Nevada Parque Nacional (Spain) for La Caldera.

### Experimental Design

A 2×2 factorial nutrient-manipulation design was implemented for 15 days in Cubillas reservoir (15 to 29 May 2012) and 19 days in La Caldera lake (31 August to 18 September 2011). The experimental setup consisted of transparent semi-spherical polyethylene bags (microcosms, 0.58 m diameter) filled with 100 L of water accurately measured with a flowmeter. The volume of microcosms was considered to be sufficient to minimize the bottle effect over the experimental incubation periods [53]–[55]. The experiments had four treatments per triplicate (control, addition of P [P treatment], of C [C treatment] and of C plus P [CP treatment]). The water used to fill in the microcosms was taken from upper layers of each water body (from 0.2 m to 0.5 m depth) with water pump, sieved through a 45-µm mesh to remove large zooplankton and mixed before and after nutrient additions. The microcosms were fixed to a PVC-tube structure and incubated *in situ* in the upper layers (from surface to ∼0.5 m depth). The C and P were added once at the beginning of the experiment with concentrations of 30 µgP L^−1^ and 290.2 µgC L^−1^, added as Na_2_HPO_4_ and sucrose, respectively. The concentrations were selected to ensure nutrient availability throughout the experiments, maintaining the N:P molar ratio of 30 in P-added treatments (i.e. P and CP) and C:P molar ratio of 300 in C-added treatments (i.e. C and CP). Thus, to maintain the N:P ratio, we added 406.4 µgN L^−1^ as NH_4_NO_3_. Nutrients were prepared by dissolving and thoroughly mixing the nutrients in double-distilled water before the addition.

### Sampling

Before the sampling, the bags (microcosms) were homogenized by gentle beating the exterior by hand. Afterwards, water was slowly pumped from each microcosm with a small water pump through a plastic tube to fill 1.5 L acid-cleaned bottles, which were transported to the laboratory for analysis.

The sampling schedule was designed to monitor the changes in the response variables to nutrient additions in three different experimental periods, defined as early, middle, and late. Early period means sampling on each of the first three days after additions in Cubillas and on each of the first four days in La Caldera; middle and late periods mean sampling, respectively, on the 5^th^ and the 14^th^ days in Cubillas, and the 14^th^ and 18^th^ days in La Caldera after additions. These periods (and the response variables measured in each) were previously fitted for each ecosystem (time as adjusted ‘within-subjects’ factor) to account for the differences in the generation times of organisms between the ecosystems, based on water temperature and plankton dynamics [56], [57]. These periods allowed us to appreciate both the rapid and delayed responses of the dependent variables to nutrient additions (as fixed ‘between-subjects’ factors).

### Physico-chemical Analyses

Temperature was measured every 0.5 m depth through the water column with YSI MPS-556 multi-parametric sensor (YSI Incorporated, OH, USA). Samples for TP and total dissolved P (TDP) were persulphate-digested and analysed as soluble reactive P applying the acid molybdate technique [58]. Samples for TN and total dissolved nitrogen (TDN) were persulphate-digested and measured as nitrate [58].

Samples for DOC were filtered by through a pre-combusted 0.7-µm Whatman GF/F glass-fiber filters, then acidified with HCl (final pH<2) and stored in darkness at 4°C until analysed [51]. DOC concentrations were measured with the high-temperature catalytic oxidation method in a Shimadzu TOC analyser (Model 5000) equipment [59].

### Functional Variables

#### Bacterial production

Bacterial production (BP) was measured following the ^3^H-thymidine method [60] modified by [61]. Briefly, to each vial (2 mL micro-centrifuge tubes) containing 1.5 mL of sample (3 replicates and 2 blanks for each experimental treatment), ^3^H-thymidine (S.A. = 48–50 Ci mmol^−1^, Perkin Elmer) was added to a final saturating concentration (12 nM in La Caldera; 20 nM in Cubillas). Vials were incubated at *in situ* temperature for 60 min in darkness. Extraction was carried out with 5% (final concentration) cold trichloroacetic acid (TCA). The tubes were centrifuged at 16,000 g, rinsed twice with 5% TCA, and measured in a scintillation counter equipped with autocalibration (Beckman LS 6000 TA). In all the calculations, data were corrected by blanks (bacteria were killed with 5% TCA before addition of the radiotracer). To convert incorporated tracers to BP expressed in C terms, we applied the conversion factors 1×10^18^ cells mol^−1^ of thymidine [62], and 2×10^−14 ^g C per cell [63].

#### Primary production

Primary production (PP) was measured following the ^14^C method [64]. Briefly, each 50-mL quartz flask (three clear and one dark from each microcosm), added with 0.37 MBq of NaH^14^CO_3_ (Specific Activity: 310.8 MBq mmol^−1^, DHI Water and Environment, Germany) was incubated in the upper layers (0.5 m depth) for 4 h symmetrically distributed at noon. PP was measured as total organic carbon, and algal excretion of organic carbon (EOC) as the organic measured in the <1 µm fraction, following the laboratory procedure of [65]. Gross primary production (GPP) was calculated as the sum of PP, standardized for 14.5 h of daytime, and planktonic community respiration (R, see below), standardized for 24 h (entire day), and thus these ratios were assumed to be constant throughout the day.

#### Community and bacterial respiration

Respiration of planktonic community (<45 µm fraction; R) and bacterial community (<0.7 µm fraction, filtrate of samples through glass-fiber filters Whatman GF/F; BR) were estimated from oxygen depletion measured with sensor-spot optodes (SP-PSt3-NAU-D5-YOP and Fibox3; PreSens GmbH, Germany). Briefly, optodes followed a two-point calibration (0 and 100% oxygen saturation), 0% point calibration was performed adding sodium sulphite (Na_2_SO_3_) to a final concentration exceeding 0.1 mg mL^−1^ and 100% by putting wet cotton wool into the closed flask to ensure a 100% O_2_-saturated water-vapour air. Samples for each fraction and experimental treatment were transferred to 25-mL quartz flasks with a sensor spot attached inside and closed with glass stoppers. Respiration rates were calculated from least-squares regressions after confirming that oxygen fitted a linear model during the first 24 h after sampling.

#### Bacterial carbon demand and availability of algal C supply

The bacterial carbon demand (BCD) was estimated as the sum of BP and BR [13], [66]. Because of the uncertainty of algal C supply during the night time both BCD and EOC were standardized for 14.5 h of day time, as in [32]. We calculated the BCD:EOC ratio to quantify the proportion of bacterial C demand met by algae-released C, and whether it underwent late period variations compared to initial conditions. The use of EOC in this ratio is based on the bacterial preference for autochthonous C [24] and experimental findings of bacterial dependence on organic C freshly released by algae in an Iberian lake [32], [67].

### Biological Variables

#### Bacterial abundance

Bacterial abundance (BA) was determined by flow cytometry technique (FACSCanto II, Becton Dickinson Biosciences, Oxford, UK) fixing 1.5 mL of sampling water with particle-free 20% (w/v) paraformaldehyde, 1% final concentration followed by liquid nitrogen frozen and stored at −80°C [68], [69]. Before being analysed, the samples were thawed and stained with Syber Green I DNA (Sigma-Aldrich) 1∶5000 final dilution of initial stock [69], [70]. Yellow-green 1-µm beads at a standard concentration (Fluoresbrite Microparticles, Polysciences, Warrington, PA, USA) were added in order to determine absolute cell concentrations [69], [71].

#### Algae and ciliate abundance and biomass

Autotrophic picoplankton (<2 µm) was checked in samples fixed with 1% paraformaldehyde, and immediately filtered through black 0.2-µm pore-size Nuclepore filters, through fluorescence microscopy with an inverted microscope (Axio Observer.A1 model, Carl Zeiss Microscopy GmbH, Germany). Phytoplankton and ciliates were preserved in glass bottles with alkaline Lugol’s solutions until analysed. Water samples (50 mL) were allowed to settle in Utermöhl chambers for 24 h and cells were counted at 400x and 1000x following [72]. At least 400 individuals of the most abundant species were counted [73], and 20 individuals per species were measured in each treatment. Biomass was estimated by approximating cell volume to their geometric shape [74], [75] and to transform it to C units following suitable conversion factors [76], [77].

### Data Analysis

For each ecosystem, the nutrient-addition effects over time were tested by two-way repeated-measures ANOVA (RM-ANOVA). Homoscedasticity (by Cochran’s and Levene’s tests), and correlation between means and standard deviations were checked for each data group in order to verify the assumptions required by RM-ANOVA. The interaction between time and resources (C, P) was tested using Pillai, Hotelling and Roy’s multivariate tests that avoid the problem of assuming compound symmetry and sphericity. When the interactive effect was significant, Fisher’s LSD *post hoc* test was applied to detect statistical significance between treatments. The effect size of nutrient additions was calculated as the quotient between the mean value of each resource-added treatment and the mean value of the control, for each response variable.

To investigate the evolution of co-limitation on bacteria over time, we estimated the type of co-limitation for each period by applying the zero-centred interaction ratio response index (“IRR”) reported by [47], but incorporating its error term (calculated by the general equation of propagation of errors) and considering zero as the threshold to distinguish among super-additive (IRR ± error>0), additive (IRR ± error = 0), and sub-additive positive responses.
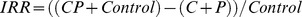
where CP, C, P, and Control represent the mean value of the response variable for each treatment.

Subsequently, the diversity of kinds of co-limitation provided by the IRR index was “summarized” in a unifying classification according to [47] and following more simplifying criteria equivalent to those reported by [46], despite that we discarded the direct use of their interaction effect index because of log-transformation biases, as discussed by [47]. We selected these two classifications because they were the only ones that enabled us to quantify the type of co- limitation, applied to bacterioplankton, and determined by the IRR values. Thus, IRR>0 indicates that the response to the simultaneous addition of both resources is higher than that of a single resource, considered as synergistic co-limitation by [46], while subdivided into three subtypes by [47] (Figure S1). IRR = 0 indicate: i) no response, when no effects of single resources are found, ii) single limitation, when only one of the single resources has a significant effect, and iii) additive (*sensu* [46]) or Independent Co-Limitation Additive (*sensu* [47]), when single resources show a significant effect, but the sum of both effects is lower than the effect of the two simultaneous resources. Finally, IRR<0 indicates that the additive effect is higher than the effect of combined resources (i.e. antagonistic responses, *sensu* [46]) or no interaction *sensu* [47]).

This unifying classification is summarized in Figure S1 as well as the abbreviations used hereafter in the text that correspond to the types of co-limitation derived only from the positive or null responses to resource additions depicted in Figure S1.

We used a SEM (structural equation model) to test whether the dynamics of inorganic and organic resources (i.e. P and C) and of potential predators (mixotrophs and ciliates) influenced BA dynamics. Our model proposed that BA is regulated by bottom-up (resources) and top-down (potential predators) controls.

All variables were assessed for normality prior to statistical analyses. The GLS→ML method was used to estimate standardized path coefficients in our model. The degree of fit of the model to the observed data was tested by χ^2^. Non-significant χ^2^ indicates that the model could be accepted. Additionally, the degree of fit was supplemented with other goodness-of-fit indices such as the Bentler–Bonnet Normed FitIndex (NFI) and the Goodness-of-Fit Index (GFI), as recommended by [78]–[80].

For all statistical tests, we assumed p<0.05 as a threshold level of acceptance, and STATISTICA 7 software (StatSoftInc, 2005) was used.

## Results

### Starting Conditions of the Experiments

Water temperature differed between the two ecosystems studied (Table 1). This was >22.5°C in the upper layers, declining gradually to 17.5°C at the bottom (6.5 m) with no stratification pattern, in Cubillas. By contrast, water temperature reached only 14°C in La Caldera, with a homogeneous vertical profile to the maximum depth (10 m).

**Table 1:** Mean values of the main physical, chemical and biological variables measured under initial conditions of the experiment.

Variable	Eutrophic ecosystem	Oligotrophic ecosystem
**Temperature** (°C)	>22.5	14
**Secchi disk** (m)	0.5	9
**TP** (µgP L^−1^)	15.13±0.13	2.18±1.11
**TDP** (µgP L^−1^)	3.73±0.33	2.12±0.70
**TN** (mgN L^−1^)	2.93±0.01	0.32±0.04
**TDN** (mgN L^−1^)	2.75±0.01	0.28±0.02
**DOC** (mgC L^−1^)	3.50±0.98	0.59±0.14
**BP** (µg C L^−1 ^h^−1^)	1.13±0.17	0.07±0.01
**R** (µg C L^−1 ^h^−1^)	6.02±2.19	0.89±0.10
**BR** (µg C L^−1 ^h^−1^)	2.19±1.57	0.33±0.09
**BA** (cells mL^−1^)	3.6×10^6^±5.3×10^5^	3.5×10^5^±2.4×10^4^
**Chl a** (µg L^−1^)	9.0±0.1	2.5±0.14
**AB** (µg C L^−1^)	824±275	18.7±3.2
**CB** (µg C L^−1^)	2±3	0.0±0.0

Abbreviations: Temperature (average water column); TP = total phosphorus; TDP = total dissolved phosphorus; TN = total nitrogen; TDN = total dissolved nitrogen; DOC = dissolved organic carbon; BP = bacterial production; R = planktonic respiration; BR = bacterial respiration; BA = bacterial abundance; Chl a = chlorophyll a; AB = algae biomass; CB = ciliate biomass.

Chemical variables, such as DOC, TP, and TN showed values between 5- and 9-fold higher in Cubillas than in La Caldera, thus representing the contrasting trophic states of the two ecosystems. This difference was further indicated by water transparency (Secchi disk depth was 18-fold higher in La Caldera) and the biotic variables, which showed higher values in Cubillas than in La Caldera (Chl *a*, 3.6-fold; algal biomass, 44-fold; BA, 10.3-fold; BP, 16.1-fold; R [<45 µm fraction], 3.8-fold; BR [<1.2 µm fraction], 8.9-fold; Table 1). Therefore, hereafter La Caldera will be designated as the oligotrophic ecosystem, and Cubillas as the eutrophic ecosystem.

Another important differentiating feature between the two ecosystems was the nanoplanktonic community composition. Thus, in the eutrophic ecosystem, the algal community was dominated by potential mixotrophic algae (up to 92% of the algal biomass) and ciliate abundance reached 27 cells mL^−1^. By contrast, in the oligotrophic ecosystem, strict autotrophic algae dominated the algal community (Chlorophyceae, up to 100%) and ciliates were not detected (Table 1).

Contrary to our expectations, the eutrophic ecosystem showed a heterotrophic nature (GPP:R<1, PP:R<1, PP:BP = 2.20) while the oligotrophic one showed the opposite scenario (autotrophic nature; GPP:R>1, PP:R>1, PP:BP = 34.47).

### Resource Variation Over the Experiments

**Figure 1:**
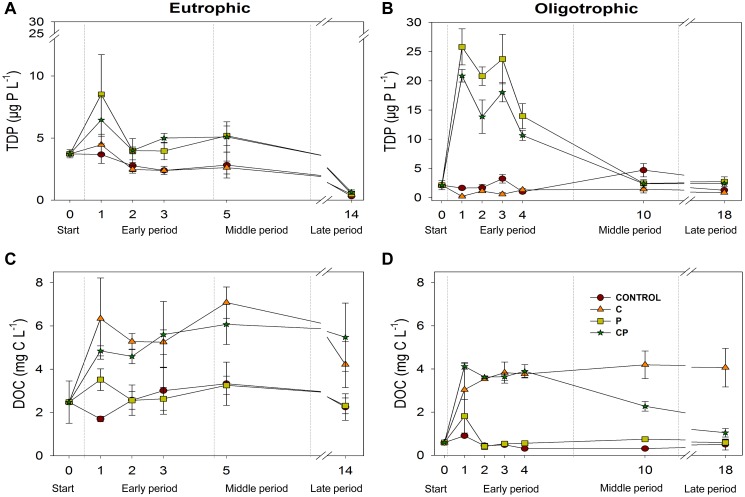
Experimental dynamics of DOC and TDP in both study ecosystems. (A) TDP in the eutrophic ecosystem (Cubillas); (B) TDP in the oligotrophic ecosystem (La Caldera); (C) DOC in the eutrophic ecosystem (Cubillas reservoir); (D) DOC in the oligotrophic ecosystem (La Caldera lake). Symbols represent mean values and error bars represent standard deviations. DOC = dissolved organic carbon. TDP = total dissolved phosphorus.

In both ecosystems, TDP followed a decreasing trend over time (RM-ANOVA, effect time p<0.05, Table S1) with a steeper decline in the P-added treatments (Figure 1A, B) while concentration of TDN did not change (RM-ANOVA, time effect p-value>0.05). By contrast, DOC showed a different pattern in each ecosystem, as it did not vary over time in the eutrophic ecosystem (RM-ANOVA, time effect p-value>0.05, Figure 1), but decreased in CP treatment over both the middle and late periods, reaching values similar to those of control in the oligotrophic ecosystem (Figure 1C, D; Table S1).

### Bacterial Production Response to Resource Addition

**Figure 2:**
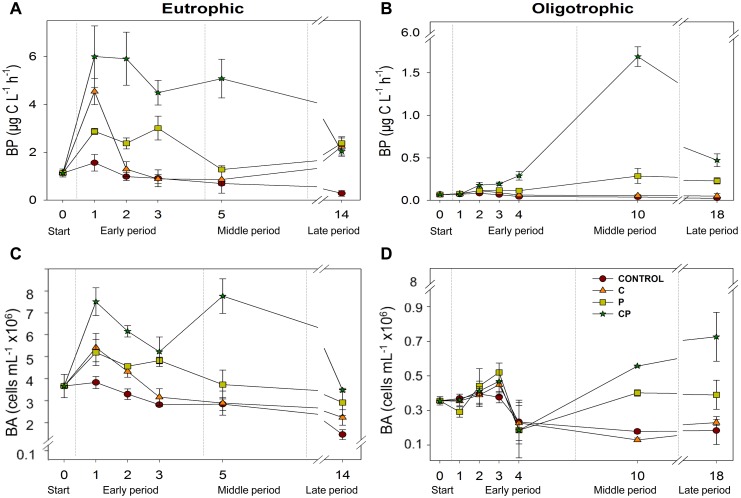
Temporal responses of bacterial production and abundance to experimental treatments in the two ecosystems. Left panels show the bacterial production (A) and abundance (C) responses in the eutrophic ecosystem, while the right panels show the same response variables (B and D, respectively) for the oligotrophic ecosystem. Symbols represent mean values and error bars represent standard deviations.

In the eutrophic ecosystem, BP responded positively to the P and C treatments over the early period. However, these responses vanished over the middle period to reappear over the late period (Figure 2A; Tables 2 and S2). Notably, BP strongly responded to CP treatment over the early and middle periods, although the magnitude of this positive response (effect size) diminished after 24 h and decreased further over the late period, when BP equalled the control or each single addition treatment (Figure 2A; Table 2). As a result, the kind of resource co-limitation for BP changed within the early period from additive (ICLA) to synergistic (*S*SL-P), which persisted over the middle period, but became antagonistic (ICLSbA) over the late period (Figure 2A; Table 3). In La Caldera, BP positively responded to P treatment after 48 h until the end of the experiment (Figure 2B; Table 4) but not to C treatment at any time (Figure 2B, Table 4). BP strongly responded to CP treatment after 48 h until the end of the experiment, with the strongest positive response at middle period (Figure 2B; Tables 4 and S2). As a result, the kind of resource co-limitation for BP was synergistic during that period.

**Table 2:** Effect size and p-value (Fisher’s *post hoc* test) for BP and BA for the eutrophic ecosystem.

			BP		BA	
Eutrophic ecosystem						
			Effect size	p-value	Effect size	p-value
**C**	Early period	24 h	2.90	**<0.01**	1.42	**<0.01**
		48 h	1.32	0.39	1.31	**<0.01**
		72 h	0.97	0.94	1.12	0.41
	Middle period		1.24	0.64	1.02	0.90
	Late period		8.08	**<0.01**	1.54	0.08
**P**	Early period	24 h	1.83	**<0.01**	1.36	**<0.01**
		48 h	2.43	**<0.01**	1.39	**<0.01**
		72 h	3.31	**<0.01**	1.72	**<0.01**
	Middle period		1.85	0.12	1.32	**<0.01**
	Late period		8.57	**<0.01**	2.01	**<0.01**
**CP**	Early period	24 h	3.83	**<0.01**	1.97	**<0.01**
		48 h	6.06	**<0.01**	1.87	**<0.01**
		72 h	4.94	**<0.01**	1.87	**<0.01**
	Middle period		7.37	**<0.01**	2.74	**<0.01**
	Late period		7.35	**<0.01**	2.41	**<0.01**

Abbreviations: BP = bacterial production; BA = bacterial abundance.

**Table 3:** IRR values and their correspondence with type of co-limitation for BP and BA for eutrophic ecosystem.

			BP			BA		
Eutrophicecosystem								
			IRR	Type ⋇	Type ∦	IRR	Type ⋇	Type ∦
**CP**	Early period	24 h	0.10±0.92	AD	ICLA	0.19±0.29	AD	ICLA
		48 h	3.30±1.26	SC	*S*SL(P)	0.17±0.13	SC	ICLSpA
		72 h	1.66±0.85	SC	*S*SL(P)	0.02±0.28	n.d.	SL(P)
	Middle period		5.28±1.52	SC	SCL	1.41±0.42	SC	*S*SL(P)
	Late period		−8.30±4.29	AA	ICLSbA	−0.15±0.51	n.d.	SL(P)
**Oligotrophic** **ecosystem**								
**CP**			**IRR**	**Type ⋇**	**Type ∦**	**IRR**	**Type ⋇**	**Type ∦**
	Early period	24 h	−0.16±0.29	n.d.	NR	0.23±0.12	n.d.	NR
		48 h	0.43±0.47	n.d.	NR	−0.06±0.26	n.d.	NR
		72 h	0.73±0.39	SC	*S*SL(P)	−0.33±0.31	AC	*A*SL(P)
		96 h	3.56±1.45	SC	*S*SL(P)	0.02±0.97	n.d.	NR
	Middle period		35.39±3.78	SC	*S*SL(P)	1.15±0.12	SC	*S*SL(P)
	Late period		8.73±4.01	SC	*S*SL(P)	1.15±0.12	SC	*S*SL(P)

IRR = Interaction Ratio Response index ± error term and its correspondence with the type of co-limitation according to a modified classification based on [46] (Type ⋇) and [47] (Type ∦) for each variable response. BP = bacterial production and BA = bacterial abundance. Abbreviations: AD = additive; SC = synergistic co-limitation; AC = antagonism co-limitation; AA = absolute antagonism; NR = no response; ICLA = independent co-limitation additive; ICLSpA = independent co-limitation super-additive; SL(P) = single limitation (P); *S*SL(P) = *synergistic* serial limitation (P); ICLSbA = independent co-limitation sub-additive; *A*SL(P) = *antagonism* serial limitation (P); n.d. = not defined.

**Table 4:** Effect size and p-value (Fisher’s *post hoc* test) for BP and BA for oligotrophic ecosystem.

			BP		BA		
Oligotrophic ecosystem							
			Effect size	p-value	Effect size	p-value	
**C**	Early period	24 h	1.05	0.90	0.96	0.79	
		48 h	1.38	0.29	0.98	0.90	
		72 h	1.36	0.41	1.19	0.21	
		96 h	1.44	0.51	0.98	0.91	
	Middle period		1.42	0.57	0.73	0.21	
	Late period		2.10	0.38	1.25	0.40	
**P**	Early period	24 h	1.07	0.84	0.79	0.18	
		48 h	1.38	0.30	1.12	0.41	
		72 h	1.80	0.09	1.38	**<0.05**	
		96 h	2.63	**<0.05**	0.80	0.40	
	Middle period		7.32	**<0.01**	2.25	**<0.01**	
	Late period		9.30	**<0.01**	2.13	**<0.01**	
**CP**	Early period	24 h	0.96	0.91	0.98	0.86	
		48 h	2.19	**<0.01**	1.04	0.77	
		72 h	2.88	**<0.01**	1.24	0.12	
		96 h	6.63	**<0.01**	0.79	0.39	
	Middle period		43.12	**<0.01**	3.13	**<0.01**	
	Late period		19.13	**<0.01**	3.95	**<0.01**	

Abbreviations: BP = bacterial production; BA = bacterial abundance.

### The Amount of Algal C Supply that Met Bacterial C Demand

In the eutrophic ecosystem, the BCD:EOC ratio yielded values >1 in all treatments (i.e. up to 16.2), suggesting that bacterial demands exceeded the supply of photosynthetic C. By contrast, in the oligotrophic ecosystem, the BCD:EOC ratio was <1 (up to 0.7) in all treatments, suggesting the opposite scenario. In each ecosystem, the values of the ratio were similar to those found for the respective starting conditions (Table S3).

### Response of the Microbial Plankton Structure to Resource Addition

In the eutrophic ecosystem, BA positively responded to all treatments with resource additions over the early period, although the magnitude of the response (effect size) to CP treatment varied over the early period; consequently, the kind of co-limitation shifted from additive (ICLA) to synergistic (ICLSpA). Over the middle period, BA responded slightly to the P and strongly to CP treatments (Figure 2C; Tables 2 and 3), generating a synergistic co-limitation (*S*SL-P). Over the late period, the response of BP to CP treatment decreased and, therefore, co-limitation was replaced by single limitation by P (Table 3).

In the oligotrophic ecosystem, BA followed a completely different dynamic, due to the lack of response to resource addition until the middle period. BA positively responded to P and CP treatments from the middle to late period (Figure 2D; Tables 3 and 4), which generated a synergistic co-limitation (*S*SL-P).

**Figure 3:**
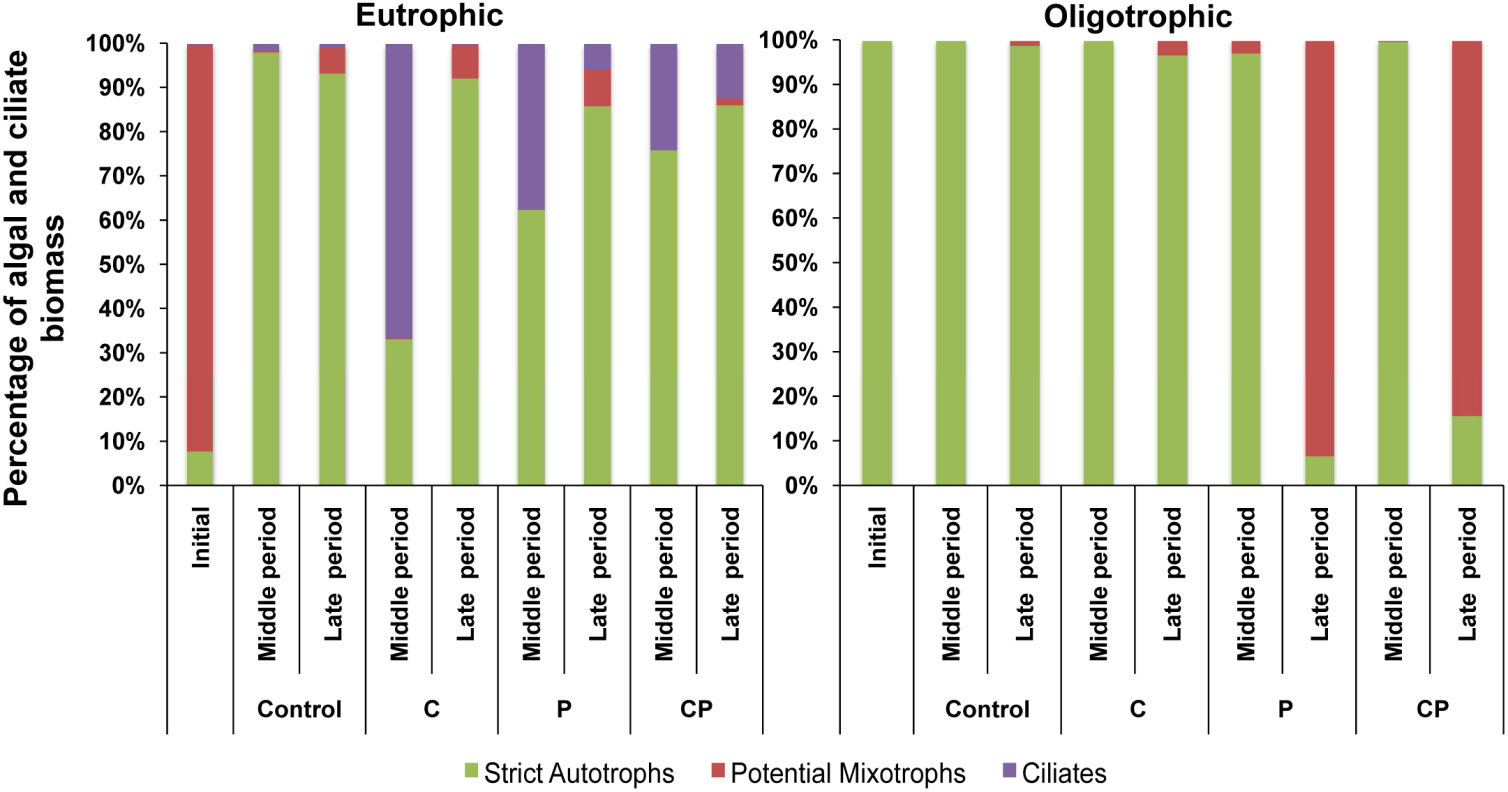
Experimental dynamics of algal and ciliate biomass in the two ecosystems. (A) eutrophic ecosystem; (B) oligotrophic ecosystem. Columns represent the percentage of mean biomass values of the different planktonic functional groups (strict autotrophs, potential mixotrophs and ciliate) over the experiment.

Figure 3 depicts the dynamics of nanoplanktonic community (autotrophic picoplankton was not detected) and its response to resource additions over the middle and late period in each ecosystem. In the eutrophic ecosystem, algal biomass showed the highest values over the late period within each treatment. In contrast to initial conditions, strict autotrophic algae dominated algal biomass over the middle and late periods, regardless of the resource addition (up to 91% of total algal biomass), although mixotrophic algae developed only slightly over the late period in all treatments (reaching up to 8.8% of algal biomass; Figure 3A). Ciliates developed in all treatments, except in control (ciliate biomass was 86.7-fold in C, 50-fold in P, and 23-fold in CP treatment) over the middle period. However, over the long period, ciliates vanished in the C treatment or sharply diminished in the P treatment (coinciding with the development of mixotrophic algae), but only slightly decreased in the CP treatment. By contrast, in the oligotrophic ecosystem, ciliates were not detected, and strict autotrophic algae became the dominant group (Figure 3B) in all experimental treatments until the middle period. Only over the late period and under P-added conditions (P and CP treatments) did mixotrophs strongly develop, reaching up to 93.5% (P treatment) or 84% (CP treatment) of algal biomass. As expected, algal biomass strongly increased under P-added conditions at the middle period (up to 155.48 µg C L^−1^ in P treatment), particularly in autotrophic algae (i.e. 7.56-fold in P and 7.60-fold in CP). However, over the late period, algal biomass decreased in all treatments.

### SEM Analysis

**Figure 4:**
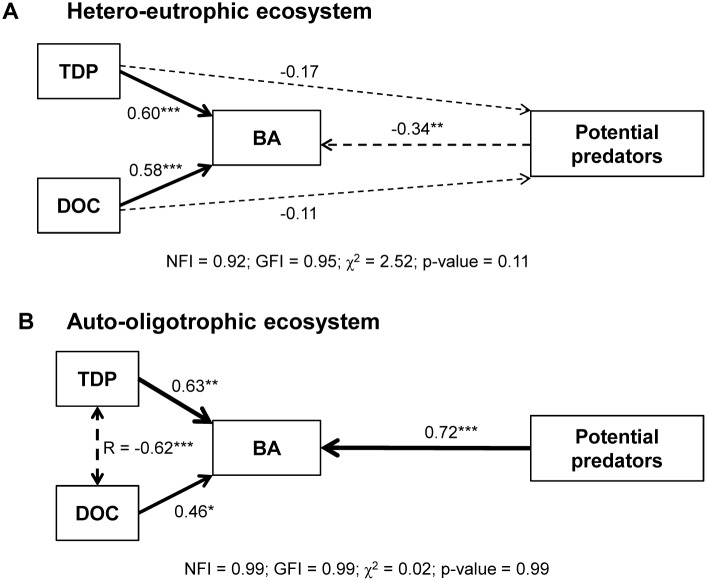
Path diagram representing the relationships between the dynamics of resources, bacterial abundance, and potential predators (mixotrophs and ciliates) for each ecosystem. One-headed arrows depict causal relationships whereas two-headed arrows depict correlations. Positive effects are indicated by solid lines and negative effects by dashed lines. Arrow widths are proportional to path coefficients. Numbers near the paths indicate standardized path coefficients. Significance path p-values are denoted with *p-value<0.05, **p-value<0.01, ***p-value<0.001, while non-significant paths are denoted with the superscript ‘n.s.’. Fit statistics (Goodness of fit index, GFI; normal fit index, NGI; χ^2^; p-value).

The SEM provided a good fit with the observed data for each ecosystem, as indicated by the non-significant χ^2^ (p-value>0.1), and by goodness-of-fit indices (NFI and GFI>0.9 in each case; Figure 4). In the eutrophic ecosystem, SEM showed significant and positive standardized path coefficients for TDP and DOC regarding their effects on BA (0.598 and 0.580 respectively; p-values<0.000) while non-significant coefficients were found regarding their direct effects on potential predators (p-values>0.05). Besides, the effect of the predators to BA was negative (-0.343; p-value<0.01). In the oligotrophic ecosystem, both resources were positively correlated (p-value>0.05), and therefore this correlation was included in the SEM, which was simpler than that of the eutrophic ecosystem. In this case, the resources affected BA but did not affect the mixotrophs. Standardized path coefficients for TDP and DOC regarding their effects on BA were positive and significant or marginally significant (TDP: 0.625, p-value<0.001; DOC: 0.460, p-value<0.05). Contrarily to the eutrophic ecosystem, the standardized path coefficient between mixotrophs and BA was positive and significant (0.716, p-value<0.001).

## Discussion

This study contributes new insights on resource co-limitation of bacterioplankton by two different resources, i.e. organic carbon (energetic resource) and phosphorus (mineral nutrient) in freshwater ecosystems, particularly in a largely unstudied ecoregion. Thus, through a modified index (adapted to bacterioplankton) based on that reported by [47], we quantified the type of resource co-limitation, and its dynamics over time. In addition, the trophic-nature gradient (i.e. autotrophy-heterotrophy axis) proves to be a key feature determining the expected types of resource co-limitation of bacteria, which are summarized in a proposed theoretical framework, based on the types found in the literature for primary producers [45]–[47], [49], [81]. On this basis, the actual types of resource co-limitation, and their dynamics, highlight the major role exerted by ecological interactions, mainly predation, altering the expected responses of bacterioplankton to limiting resources.

### The Resource (C vs. P) that Mainly Limited Bacterioplankton

Based on the IRR index (which measures the relative magnitude of the bacterial responses to joint C and P addition compared to single-resource addition), our experimental test of resource limitation consistently showed that both resources (C and P) co-limited bacterioplankton in two ecosystems with contrasting trophic states. In this regard, the SEM for each ecosystem showed a positive path-analysis effect of both resource dynamics on bacterioplankton (BA). Therefore, our results extend to bacterioplankton the prevalence of resource co-limitation found for primary producers [45], [47] and agree with the current criticism regarding the broad relevance of Liebig’s law of the minimum for more complex organizational levels than individual organisms [81], [82].

Through an analysis of the bacterial response to single-resource additions, we distinguish which of them mainly limited bacteria in each ecosystem. Overall, our results regarding BP and BA responses contradicted our initial hypothesis and the conclusions of [32] that C (not P) was the main limiting resource for bacteria in oligotrophic ecosystems. That is, in our study, bacteria did not respond to C but did to P treatments in the oligotrophic ecosystem. Nevertheless, our results agree with the conclusions of [32] stating that mainly P and secondarily C were co-limiting resources under eutrophic conditions because, in the eutrophic ecosystem of our study, bacteria responded to P and C treatments. Notably, the response to the P treatment in most cases had a greater magnitude (effect size) than in C treatment. These interpretations of resource limitation are also supported by the values shown by the BCD:EOC ratio, a proxy for bacterial limitation of autochthonous C. This proxy measures the ability of algal C (EOC) to meet bacterial demands for C, based on the reported bacterial preference for this C source [25], [26], [32]. Thus, the BCD:EOC ratio indicates that bacteria were limited by autochthonous C in the eutrophic ecosystem (BCD:EOC>1), but not in the oligotrophic lake (BCD:EOC<1) at starting conditions and over the late period.

**Figure 5:**
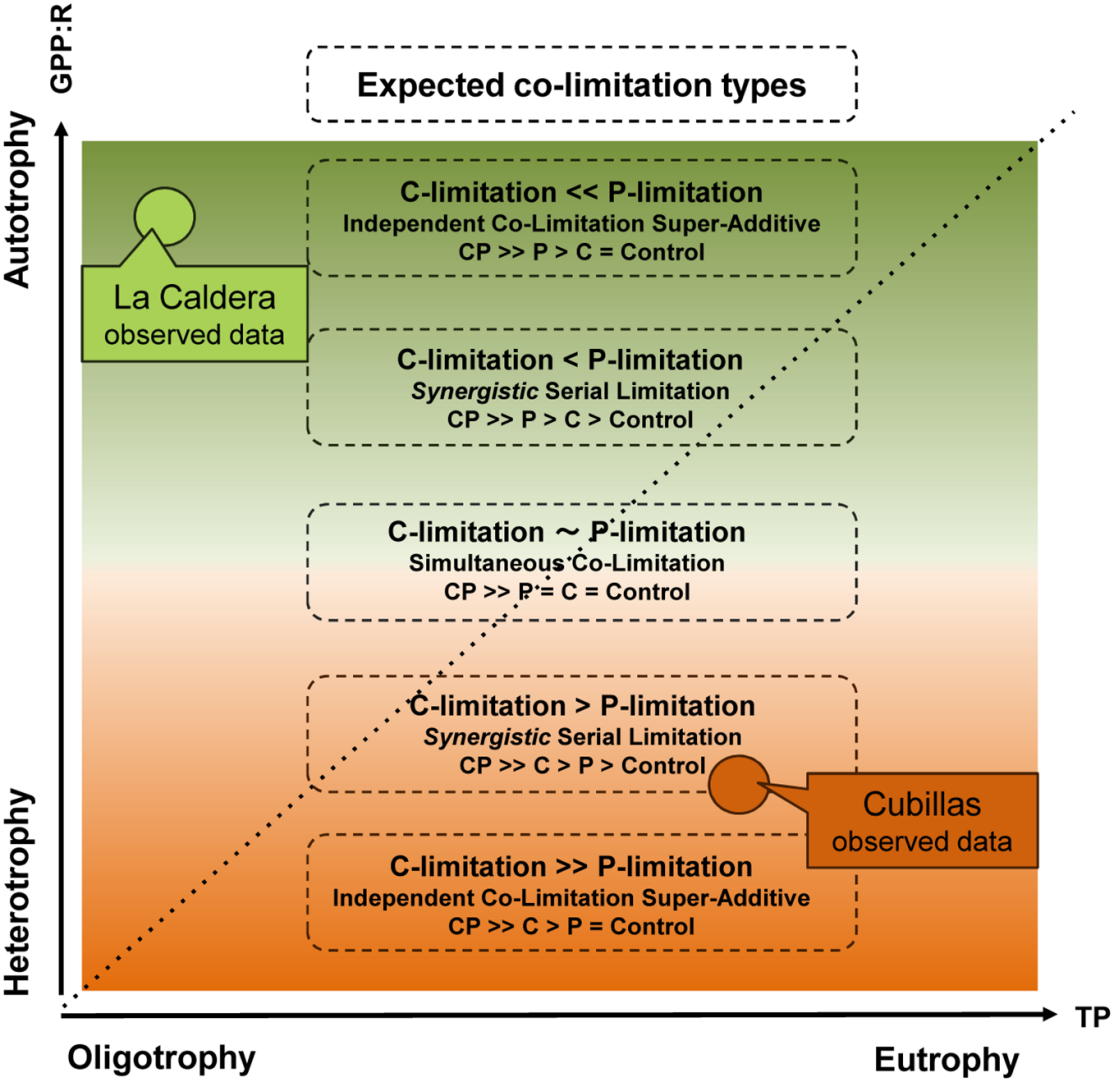
Theoretical diagram of expected types of C and P co-limitation of bacteria based on the trophic-nature gradient. The diagram represents the different types of expected co-limitation according to the trophic-nature gradient (Y-axis), indicated by the colour gradient from dark brown (maximum heterotrophy) to dark green (maximum autotrophy). The diagonal dashed line arrow crossing the diagram represents the trophic-state gradient (X-axis) in the sense of more autotrophy more eutrophy, as found in [32], which does not reflect the major role of trophic-nature gradient found in the present study. The squares with dashed lines include: (i) the single resource expected to be more limiting, according the trophic nature axis; (ii) the name of the expected co-limitation type *sensu* [47], and its meaning as effects of the treatments. The circles represent the position found for the ecosystems according to both axes; brown circle is the hetero-eutrophic ecosystem (Cubillas), green circle is the auto-oligotrophic ecosystem (La Caldera).

The discrepancies regarding the expected bacterial limitations can be explained by the trophic nature (i.e. autotrophy-heterotrophy) of the ecosystems studied. Thus, the eutrophic ecosystem was heterotrophic, and the BCD:EOC ratio indicated that the EOC was not sufficient to support bacterial demands; by contrast, the oligotrophic lake was autotrophic, and the BCD:EOC ratio showed the opposite scenario of (autochthonous) C sufficiency. In this regard, the trophic nature of the two ecosystems, measured as GPP:R or PP:R ratios, deviated from the expected pattern for the Mediterranean inland waters with respect to the trophic nature linked to the trophic gradient (i.e. increasing autotrophy towards eutrophy, [32]). It is important to point out that the trophic nature of the ecosystems studied was appropriately estimated by the quantification of respiration in the two major fractions of the planktonic community (i.e. community and picoplankton) and the lack of autotrophic picoplankton in both ecosystems. Therefore, we consider that our estimation of the trophic nature of the two ecosystems through the GPP:R ratio, from the direct measurements of respiration of both fractions, is more realistic than if it had been estimated from PP:BP or BR:BP ratios with BR determined from empirical models, as in [32]. In this sense, [83] underline the importance of the evaluation of both primary production and key heterotrophic processes (e.g. respiration) necessary to define autotrophic and heterotrophic states that expand the concept of the trophic state in aquatic ecosystems. In this regard, the correspondence between (autochthonous) C sufficiency for bacteria in the autotrophic ecosystem and the opposite scenario in the heterotrophic ecosystem in our study lead us to conclude that the trophic-nature gradient, rather than trophic-state gradient (i.e. oligotrophy-eutrophy axis, based in TP), can have a major role determining the resource-limitation patterns. Consequently, based on the trophic-nature gradient, we propose a theoretical framework (Figure 5) to distinguish among the main types of co-limitation expected for bacteria and described in the literature for primary producers [45]–[47]. In addition, we propose a unifying classification from the diversity of types of co-limitation reported in the literature, as summarized in Figure S1. In this context, our findings disagree with the conclusions of [32] because the relation between the trophic-nature and trophic-state gradients (i.e. more autotrophy with eutrophy) does not necessarily hold in the Mediterranean ecoregion, as our more accurate results regarding autotrophy or heterotrophy reveals. Additionally, our experimental findings confirm the intersystem variability existing in the Mediterranean ecoregion, even at local scale (e.g. oligotrophic-autotrophic vs. eutrophic-heterotrophic ecosystems).

### The Potential Effect of Ecological Interactions on the type of Co-limitation Over Time

With respect to the fastest and most responsive bacterial variable to resource addition (i.e. BP), our results fit the expected theoretical types of co-limitation depicted in Figure 5, although with a lesser degree of synergism. Thus, in the eutrophic-heterotrophic ecosystem, the maximum BP response to CP treatment (over the early period) generated an additive co-limitation (CP>C>P>Control; ICLA), partially deviating from the theoretical expectations of synergistic co-limitation (CP>>C>P>Control; ICLSpA; Figures 5 and S1). Plausible mechanisms explaining these deviations are ecological interactions that also regulate the bacterioplankton, such as predation, the role of which as an external force to physiological limitations in promoting antagonistic-like co-limitations was also discussed by [46]. In our study, this interpretation is supported by the negative path-analysis effect that the dynamics of mixotrophic algae and ciliates exert on the structural bacterioplankton variable (BA). Moreover, this interpretation also agrees with the delay in the transference of the predominant type of co-limitation from function (BP, cell division) to structure (BA); thus *S*SL-P emerged for BP over the early period, but was delayed for BA until the middle period. The uncoupling between structural and functional variables has widely been described in the literature as evidence of top-down regulation (from [7]), also shown through empirical models (see [8]) describing how regulation of bacteria can shift over time, with a relative stronger regulation by predation at early stages. In this view, the role of nutrient regeneration mediated by grazers can be a major mechanism promoting the maintenance of predator-prey system [8], [84], which accounted for the bacterial responses found over middle and late periods. Thus, it is remarkable that the type of synergistic co-limitation found for BP in the eutrophic-heterotrophic ecosystem shifted from *S*SL-P (CP>P>C = Control) to SCL (CP>>P = C = Control, Figure S1) over the middle period, and even that synergistic co-limitation became absolute antagonistic co-limitation (ICLSbA) over the late period, an infrequent type of co-limitation strongly influenced by extrinsic forces [46].

In the oligotrophic-autotrophic ecosystem, the fact that the synergistic co-limitation for both BP and BA was delayed until the middle period, when TDP was already exhausted, may be due to the lower water temperature instead of to a severe top-down control. It is known that low temperature (<15°C) can limit bacterial productivity [85] and diminish the positive response of bacteria to limiting nutrient [86], [87], while mixotrophs and ciliates (the main predators on bacteria in this ecosystem, [41], [52]) were minor nanoplanktonic components from initial conditions to middle period. In this respect, the SEM shows a positive path-analysis effect of mixotrophic algal dynamics on BA, supporting the idea that the predatory control was negligible. This fact could promote or, at least not hinder, the coupling of synergistic co-limitation (*S*SL-P) for BA and BP over the middle period.

In conclusion, our work shows the predominance of carbon and phosphorus co-limitation of heterotrophic bacteria, and that the types of co-limitation shifted over time, partially deviating from the theoretical expectations. This deviation may be due to modulation by extrinsic ecological forces, such as predatory control. Our results indicate the difficulty of accurate predicting which resource mainly limits organisms based only on their physiological limitations. Our approach helps explain which type of resource co-limitation actually occurs in ecosystems with contrasting trophic states, underlining the preponderant role of the trophic nature over the trophic state.

### Ecological Implications

Based on our results, the nature of the resource that mainly limits bacteria depending on the trophic nature of their host ecosystems is an important trait that may determine whether freshwaters act as a net C source or sink. Thus, eutrophic-heterotrophic ecosystems in which heterotrophic processes predominate (GPP<R) and with bacteria capable of consuming C (alone or combined with P) may act as net C sources through bacterial respiration. By contrast, oligotrophic ecosystems where autotrophic processes predominate (GPP>R) and with bacteria unable to respond to C alone, may act as a net C sink. Moreover, in the oligotrophic-autotrophic ecosystems, inputs of organic carbon and inorganic nutrients would not alter their behaviour as net C sinks, because their autotrophic feature (GPP>R) would remain unchanged even under these resource-enriched conditions. Therefore, in these autotrophic ecosystems, the contribution of bacteria in C accumulation may be lower than in the eutrophic-heterotrophic ecosystems, due to the high GPP:R ratio, showing the major contribution of primary producers. Our work is in line with the increasingly recognized role of freshwater ecosystems in the global C cycle [88], and highlights the need for more attention to be placed on the heterotrophic microbial compartment because of their key role in freshwaters, particularly in the heterotrophic ecosystems.

Remarkably, the prevalence of C and P co-limitation of bacteria in both types of ecosystems indicates that bacterioplankton is highly responsive to human increases in mineral nutrients and organic matter. This increment, therefore, may alter not only bacterial structure and function [89], but also scale to ecosystem functioning [90], [91], ultimately deteriorating the ecosystem services that they provide [10], [42].

## Supporting Information

Figure S1 **Flow chart of logical tests used to categorize the types of resource co-limitation for bacteria.** The proposed types from the positive responses to both resources are depicted according to a modified classification based on [47] (black abbreviations) and [46] (blue text). See text for more details. Y or N correspond to “yes” or “no” (logical true or false). Synergistic co-limitation (SC) indicates that the response to the CP treatment is greater than the sum of the response to C alone and P alone; additive co-limitation (AD) indicates that the response to CP is equal to the sum of the response to C alone and P alone; antagonistic co-limitation (AC) indicates that the response to CP is greater than that of either C or P alone, but not their sum; absolute antagonism (AA) indicates that the CP response is less than that of either C or P alone. ICLSpA = Independent Co-Limitation Supper-Additive; *S*SL = *Synergistic* Serial Limitation; SCL = Simultaneous Co-Limitation; ICLA = Independent Co-Limitation Additive; SL = Single Limitation; NR = No Response; ICLSbA = Independent Co-Limitation Sub-Additive; *A*SL = *Antagonistic* Serial Limitation; NI = Negative Interaction.
(TIFF)

Table S1 **Results from two-way RM-ANOVA and multivariate tests of Pillai, Hotelling, and Roy for dissolved organic carbon, total dissolved phosphorus and total dissolved nitrogen for both aquatic ecosystems.** F values with their corresponding degrees of freedom and significance levels (p) are shown for each resource treatment and resource treatment × time in each variable. DOC = dissolved organic carbon; TDP = total dissolved phosphorus; TDN = total dissolved nitrogen.
(PDF)

Table S2 **Results from two-way RM-ANOVA and multivariate tests of Pillai, Hotelling, and Roy of bacterial production and bacterial abundance for both aquatic ecosystems.** F values with their corresponding degrees of freedom and significance levels (p) are shown for each resource treatment and resource treatment × time in each variable. BP = bacterial production; BA = bacterial abundance.
(PDF)

Table S3 **Results from two-way ANOVA of the bacterial carbon demands: excretion of organic carbon ratio for both ecosystems.** F values with their corresponding degrees of freedom and significance levels (p) are shown for each resource treatment and resource treatment × time in the bacterial carbon demand: excretion of organic carbon (BCD:EOC).
(PDF)
