# Supplementary figures and images for: Quantification of Carbon and Phosphorus Co-Limitation in Bacterioplankton: New Insights on an Old Topic
Source: PLoS One. 2014 Jun 11;9(6):e99288. doi: 10.1371/journal.pone.0099288 (PMC4053443; doi:10.1371/journal.pone.0099288)

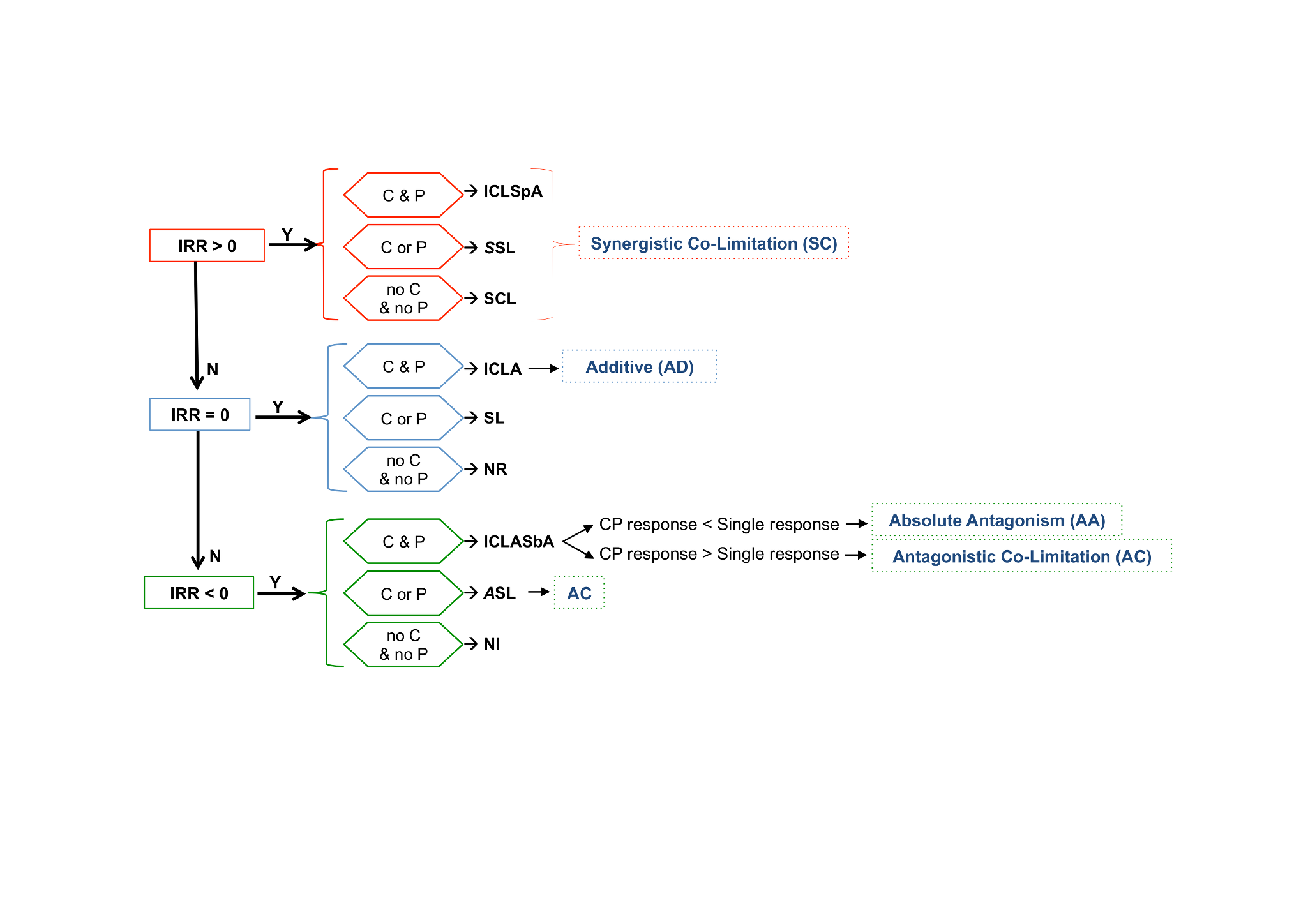

Supplement: Figure S1 — Flow chart of logical tests used to categorize the types of resource co-limitation for bacteria. The proposed types from the positive responses to both resources are depicted according to a modified classification based on [47] (black abbreviations) and [46] (blue text). See text for more details. Y or N correspond to “yes” or “no” (logical true or false). Synergistic co-limitation (SC) indicates that the response to the CP treatment is greater than the sum of the response to C alone and P alone; additive co-limitation (AD) indicates that the response to CP is equal to the sum of the response to C alone and P alone; antagonistic co-limitation (AC) indicates that the response to CP is greater than that of either C or P alone, but not their sum; absolute antagonism (AA) indicates that the CP response is less than that of either C or P alone. ICLSpA = Independent Co-Limitation Supper-Additive; SSL = Synergistic Serial Limitation; SCL = Simultaneous Co-Limitation; ICLA = Independent Co-Limitation Additive; SL = Single Limitation; NR = No Response; ICLSbA = Independent Co-Limitation Sub-Additive; ASL = Antagonistic Serial Limitation; NI = Negative Interaction. (TIFF) [file pone.0099288.s001.tiff]
