# Supplementary material for: Quantification of Carbon and Phosphorus Co-Limitation in Bacterioplankton: New Insights on an Old Topic
Source: PLoS One. 2014 Jun 11;9(6):e99288. doi: 10.1371/journal.pone.0099288 (PMC4053443; doi:10.1371/journal.pone.0099288)
Supplement: Table S1 — Results from two-way RM-ANOVA and multivariate tests of Pillai, Hotelling, and Roy for dissolved organic carbon, total dissolved phosphorus and total dissolved nitrogen for both aquatic ecosystems. F values with their corresponding degrees of freedom and significance levels (p) are shown for each resource treatment and resource treatment × time in each variable. DOC = dissolved organic carbon; TDP = total dissolved phosphorus; TDN = total dissolved nitrogen. (PDF) [file pone.0099288.s002.pdf]

Table S1. Results from two-way RM-ANOVA and multivariate tests of Pillai, Hotelling, and Roy for DOC, TDP and TDN for both aquatic ecosystems.

		DOC		TDP		TDN	
**Eutrophic ecosystem**							
		$F_{1,12}$	p-value	$F_{1,7}$	p-value	$F_{1,12}$	p-value
	P	3.30	0.093	171.91	<0.001	46.71	<0.001
	C	155.55	<0.001	11.53	<0.001	0.13	0.724
	P × C	0.04	0.8421	18.90	<0.001	1.96	0.186
		$F_{4,9}$	*p*	$F_{4,4}$	*p*	$F_{4,9}$	*p*
	Time	4.64	<0.001	80.39	<0.001	41.53	<0.001
	Time × P	2.56	0.110	10.30	<0.001	10.28	<0.001
	Time × C	1.88	0.197	4.51	0.086	1.18	0.379
	Time × P × C	3.12	0.072	9.90	<0.050	1.44	0.296
**Oligotrophic ecosytem**							
		$F_{1,8}$	p-value	$F_{1,8}$	p-value	$F_{1,8}$	p-value
	P	707.55	<0.001	2397.98	<0.001	111.44	<0.001
	C	31.43	<0.001	106.54	<0.001	2.80	0.169
	P × C	6.42	<0.001	21.77	<0.001	0.26	0.619
		$F_{5,4}$	*p*	$F_{5,4}$	*p*	$F_{5,4}$	*p*
	Time	376.63	<0.001	376.63	<0.001	1.034	0.500
	Time × P	462.35	<0.001	462.35	<0.001	1.37	0.389
	Time × C	6.15	0.051	6.15	0.051	0.85	0.575
	Time × P × C	16.20	<0.01	16.20	<0.01	0.23	0.928

F values with their corresponding degrees of freedom and significance levels (p) are shown for each resource treatment and resource treatment × time in each variable. DOC = dissolved organic carbon; TDP = total dissolved phosphorous; TDN = total dissolved nitrogen.
