# Supplementary material for: Quantification of Carbon and Phosphorus Co-Limitation in Bacterioplankton: New Insights on an Old Topic
Source: PLoS One. 2014 Jun 11;9(6):e99288. doi: 10.1371/journal.pone.0099288 (PMC4053443; doi:10.1371/journal.pone.0099288)
Supplement: Table S2 — Results from two-way RM-ANOVA and multivariate tests of Pillai, Hotelling, and Roy of bacterial production and bacterial abundance for both aquatic ecosystems. F values with their corresponding degrees of freedom and significance levels (p) are shown for each resource treatment and resource treatment × time in each variable. BP = bacterial production; BA = bacterial abundance. (PDF) [file pone.0099288.s003.pdf]

Table S2. Results from two-way RM-ANOVA and multivariate tests of Pillai, Hotelling, and Roy of BP and BA for both aquatic ecosystems.

	BP		BA	
**Eutrophic ecosystem**				
	$F_{1,12}$	p-value	$F_{1,12}$	p-value
P	356.19	<0.001	219.37	< 0.001
C	229.29	<0.001	96.65	< 0.001
P × C	30.69	<0.001	15.66	< 0.001
	$F_{4,9}$	p-value	$F_{4,9}$	p-value
Time	39.16	<0.001	87.78	< 0.001
Time × P	17.02	<0.001	7.21	< 0.001
Time × C	23.02	<0.001	7.78	< 0.001
Time × P × C	40.82	<0.001	10.78	< 0.001
**Oligotrophic ecosystem**				
	$F_{1,8}$	p-value	$F_{1,8}$	p-value
P	973.20	<0.001	76.85	<0.001
C	447.13	<0.001	13.46	<0.050
P × C	348.83	<0.001	11.93	<0.050
	$F_{5,4}$	p-value	$F_{5,4}$	p-value
Time	1114.31	<0.001	7.42	< 0.050
Time × P	1042.80	<0.001	20.77	< 0.001
Time × C	621.60	<0.001	1.58	0.339
Time × P × C	513.86	<0.001	7.83	< 0.050

F values with their corresponding degrees of freedom and significance levels (p) are shown for each resource treatment and resource treatment × time in each variable. BP = bacterial production; BA = bacterial abundance.
