# Supplementary material for: Quantification of Carbon and Phosphorus Co-Limitation in Bacterioplankton: New Insights on an Old Topic
Source: PLoS One. 2014 Jun 11;9(6):e99288. doi: 10.1371/journal.pone.0099288 (PMC4053443; doi:10.1371/journal.pone.0099288)
Supplement: Table S3 — Results from two-way ANOVA of the bacterial carbon demands: excretion of organic carbon ratio for both ecosystems. F values with their corresponding degrees of freedom and significance levels (p) are shown for each resource treatment and resource treatment × time in the bacterial carbon demand: excretion of organic carbon (BCD:EOC). (PDF) [file pone.0099288.s004.pdf]

Table S3. Results from two-way ANOVA of the BCR:EOC ratio for both ecosystems.

			BRC:EOC	
**Eutrophic ecosystem**				
			$F_{1,12}$	p
	Late period	P	9.51	<0.01
		C	0.38	0.544
		P × C	8.69	<0.01
**Oligotrophic ecosystem**				
			$F_{1,8}$	p
	Late period	P	155.20	<0.001
		C	2.33	0.164
		P × C	2.44	0.156

F values with their corresponding degrees of freedom and significance levels (p) are shown for each resource treatment and resource treatment × time in the bacterial requirement for carbon:excretion of organic carbon (BRC:EOC).
